# Supplementary material for: Mosquito and primate ecology predict human risk of yellow fever virus spillover in Brazil
Source: Philos Trans R Soc Lond B Biol Sci. 2019 Aug 12;374(1782):20180335. doi: 10.1098/rstb.2018.0335 (PMC6711306; doi:10.1098/rstb.2018.0335)
Supplement: Supplementary Materials [file rstb20180335supp1.pdf]

# Supplementary Material

Supplement to "Mosquito and primate ecology predict human risk of yellow fever virus spillover in Brazil"

*Marissa L. Childs*, Nicole Nova, Justine Colvin, Erin A. Mordecai*

**To whom correspondence should be addressed: marissac@stanford.edu*

## Contents

**S1 Spillover model details 5**
S1.1 Model details . . . 5
S1.2 Data . . . 5

**S2 Mechanistic sub-model details 5**
S2.1 Mosqito density . . . 5
S2.1.1 Methods . . . 5
S2.1.2 Data . . . 5
S2.1.3 Results . . . 8
S2.2 Mosquito seasonality . . . 8
S2.2.1 Methods . . . 8
S2.2.2 Data . . . 8
S2.2.3 Results . . . 8
S2.3 Mosquito survival . . . 11
S2.3.1 Methods . . . 11
S2.3.2 Data . . . 11
S2.3.3 Results . . . 12
S2.4 Mosquito infectiousness . . . 12
S2.4.1 Methods . . . 12
S2.4.2 Data . . . 12
S2.4.3 Results . . . 12
S2.5 Mosquito dispersal . . . 13
S2.5.1 Methods . . . 13
S2.5.2 Data . . . 13
S2.5.3 Results . . . 13

**S3 Phenomenological primate dynamics details 16**
S3.1 Methods . . . 16
S3.2 Data . . . 16
S3.3 Results . . . 16

**S4 Model-data comparison details 16**
S4.1 Methods . . . 16
S4.2 Data . . . 16
S4.3 Results . . . 16

**S5 Boosted regression tree 17**
S5.1 Methods . . . 17
S5.2 Data . . . 18
S5.3 Results . . . 18

**S6 References 21**

S6.1 Table References . . . . . . . . . . . . . . . . . . . . . . . . . . . . . . . . . . . . . . . . . 21
S6.2 Main Text References . . . . . . . . . . . . . . . . . . . . . . . . . . . . . . . . . . . . . . 22

## List of Tables

Table S1 Data sources for spillover model, including information on the spatial resolution and range, temporal cadence and range, and use of the data. . . . . . . . . . . . . . . . . . . . . 6
Table S2 Data sources for species distribution model, including information on the spatial resolution and range, temporal cadence and range, and use of the data. . . . . . . . . . . . . . . . . . . 7
Table S3 Coefficients from logistic regression of seasonal relative mosquito abundance on current and lagged relative rainfall. . . . . . . . . . . . . . . . . . . . . . . . . . . . . . . . . . . . . 8
Table S4 Model-spillover comparison results. AIC, logistic regression coefficient, logistic regression p-value, and Bonferroni adjusted logistic regression p-value are all reported from a logistic regression of spillover on model estimates and AUC is reported from the receiver operating characteristic curve from predicting spillover with model estimates. . . . . . . . . . . . . . . 17
Table S5 Model-case comparison results. Number of reported cases given spillover predicted by risk metrics and vaccine coverage. We also calculate Spearman's rank correlation coefficient for number of cases and risk metrics. . . . . . . . . . . . . . . . . . . . . . . . . . . . . . . 17
Table S6 Data sources for boosted regression tree analysis, including information on the spatial resolution and range, temporal cadence and range, and use of the data. . . . . . . . . . . . . 19
Table S7 Comparison of predictive deviance across boosted regression tree parameters. . . . . . . 21

## List of Figures

Figure S1 Partial dependence plots of covariates used in species distribution model. Histograms show the distribution of pixels at each covariate value (left axis) and solid lines show the marginal effects of covariate on model prediction (right axis). Covariates with flat marginal effects were identified as unimportant for model prediction. LST Land Surface Temperature, EVI Enhanced Vegetation Index . . . . . . . . . . . . . . . . . . . . . . . . . . . . . . . 9
Figure S2 Predicted distribution of vectors from species distribution model, with black points indicating presence locations. Color indicates probability of occurence from highest (green) to lowest (peach). . . . . . . . . . . . . . . . . . . . . . . . . . . . . . . . . . . . . . . . . . 10
Figure S3 Comparison of mosquito seasonality model to data. Colored lines show data from field studies and dashed black lines show model estimates. Each panel is labeled by the species and study. . . . . . . . . . . . . . . . . . . . . . . . . . . . . . . . . . . . . . . . . . . . . . 11
Figure S4 At each temperature where experiments were performed, we plot observations of mosquito biting that resulted in transmission (1) or no transmission (0) on the y-axis and number of days post infectious blood meal on the x-axis in blue points. The black solid line is the modeled probability of mosquito infectiousness, which takes into account both vector competence and a log-normally distributed EIP. Each panel is labeled by the temperature it represents in degrees Celsius. . . . . . . . . . . . . . . . . . . . . . . . . . . . . . . . . . 13
Figure S5 Each of the parameters govering mosquito infectiousness is modeled as temperature dependent. Vector competence determines the horizontal asymptote of mosquito infectiousness, time to 50% infectious determines the point at which mosquito infectiousness is 50% of the way to vector competence, and standard deviation is the the standard deviation of the exponent of the log-normal distrbution. Solid black lines show model estimates. Blue bars show the range of observed temperatures in lab studies and red bars show range of monthly average temperatures observed in Brazil. . . . . . . . . . . . . . . . . . . . . . . . . . . . . . . . . 14
Figure S6 Comparison of dispersal kernel estimate to data. Points are labaled by their trapping location letter from [14]. Red line indicates estimated number of mosquitoes caught at each location from the negative exponential dispersal model when accounting for trapping effort in each sampling location. 95% confidence interval from negative binomial distribution shown in grey shading. . . . . . . . . . . . . . . . . . . . . . . . . . . . . . . . . . . . . . . . . . 15
Figure S7 Comparison of predictive deviance across tree complexity and learning rate. Cross validation residual deviance was used to select the optimal number of trees up to 5000 for each set of parameters. . . . . . . . . . . . . . . . . . . . . . . . . . . . . . . . . . . . . . . . 18

Figure S8 Partial dependence plots of all variables included in the boosted regression tree analysis with histograms showing the distribution of each covariate (left axis) and solid black line showing marginal effect of covariate on model prediction of spillover (right axis). . . . . . . . 20

# S1 Spillover model details

## S1.1 Model details

We approximate environmental risk by discretizing to months rather than continuous time and using a sum over the current month and previous three months:

$$b(\vec{y},t)\beta_h(\vec{y},t)\sum_{\tau=t-3}^{\tau=t}\int_{\vec{x}}\rho_v(\vec{x},\tau)b(\vec{x},\tau)\beta_p(\vec{x},\tau)\kappa(\vec{x},\tau)EIP(T(\vec{x})),t-\tau)s(T(\vec{x})),t-\tau)d(||\vec{y}-\vec{x}||)dxd\tau.$$

Here, $\rho_v(\vec{x},\tau)$ is the density of sylvatic vectors, $b(\vec{x},\tau)$ is the biting rate of vectors, $\beta_p(\vec{x},\tau)$ is the probability of biting a non-human primate, $\kappa(\vec{x},\tau)$ is the non-human primate infection prevalence, $EIP(T(\vec{x})),t-\tau)$ is the probability the vector has completed the extrinsic incubation period and has become infectious, $s(T(\vec{x})),t-\tau)$ is the probability of vector survival, and $d(||\vec{y}-\vec{x}||)$ is vector dispersal. For more detailed variable definitions see Table 1 (Main Text). The model is run in Google Earth Engine (1). The built-in functionality of Google Earth Engine allows for calculations between data sources of differing scales and projections by performing the calculations for a specified output pixel with specified projection and scale. We use the default scale: 1 km x 1 km pixels.

## S1.2 Data

The data used for the spillover model are described in Table S1.

# S2 Mechanistic sub-model details

## S2.1 Mosqito density

### S2.1.1 Methods

We fit species distributions models to combined *Haemagogus janthinomys*, *Hg. leucocelaunus*, and *Sabethes chloropterus* mosquito occurrence data using sampling-bias corrected background points (2). We fit the models using the `maxnet` package in R (3) with a range of regularization parameters (0.5, 1.0, 1.5, 2.0, 2.5, 3.0, 3.5, 4.0, 4.5, 5.0, 6.0, 7.0, 8.0, 9.0, 10.0, 12.5, 15.0, 17.5, 20.0) and feature classes (linear; linear and hinge; linear and quadratic; linear, hinge, and quadratic; linear and product; linear, quadratic, and product; linear, hinge, and product; and linear, hinge, quadratic, and product) and select the model with the lowest small-sample-size corrected Akaike information criterion (AICc).

We use a complementary log-log (cloglog) transform to estimate occurrence probability (4), and calculate mosquito density from occurrence probability ($p$) as $\log(1/(1-p))$ (5).

### S2.1.2 Data

Covariates are extracted using Google Earth Engine (1). The data used for the species distribution model are described in Table S2.

Occurrence points are from Global Biodiversity Information Facility (6–8) and a search of the literature. We searched in Scopus using the search term "ALL ( ( haemagogus OR sabethes ) AND ( trap* OR collect* OR field OR site OR sample ) )" on June 22, 2018. We searched in Web of Science using the search term "haemagogus OR sabethes" on July 19, 2018. We then limited to papers that caught *Hg. janthinomys*, *Hg. leucocelaunus*, or *Sabethes chloropterus* mosquitoes in South America and reported the GPS location of

Table S1: Data sources for spillover model, including information on the spatial resolution and range, temporal cadence and range, and use of the data.

| **Name** | **Source** | **Spatial Resolution (Spatial Range)** | **Temporal Cadence (Temporal Range)** | **Use** |
|---|---|---|---|---|
| Forest cover | MODIS MOD44B V006 [1] | 250 m (Global) | Yearly (2000-2016) | Used to approximate reservoir-vector and human-vector contact. Assumed 2017 and 2018 identical to 2016 for model estimates of 2017 and 2018. |
| Primate ranges | IUCN [2] | NA (Global) | Static (NA) | Limited to species in *Ateles*, *Aotus*, *Alouatta*, *Saimiri*, *Cebus*, *Callicebus*, *Callithrix*, *Saguinus*, and *Lagothrix* genera [3]. Where no species range maps occurred, reservoir-vector contact rate set to zero. |
| Precipitation | TRMM 3B43 [4] | 0.25 arc degrees (Global) | Monthly (Jan 1998 - Sep 2018) | Used to drive seasonal vector abundance through logistic model fit to field data. NOTE: Used TRMM/3B43V7 image collection available on Google Earth Engine. |
| Human population | CIESIN GPWv4 [5] | 30 arc seconds (Global) | 5 years (2000 - 2020) | Linearly interpolated between 5 year population estimates to determine yearly population estimate. Scales immunological risk to estimate population-scaled risk. NOTE: Used CIESIN/GPWv4/unwpp-adjusted-population-count image collection available on Google Earth Engine. |
| Air temperature | GLDAS-2.1 [6] | 0.25 arc degrees (Global) | 3 hours (Jan 2001 - Oct 2018) | Aggregate to monthly average air temperature. Monthly average air temperature used in estimating vector survival and infectiousness using mechanistic trait models. NOTE: Used NASA/GLDAS/V021/NOAH/G025/T3H image collection available on Google Earth Engine. |
| Vaccine coverage | Freya Shearer (personal communication) | Municipality (South America and Africa) | yearly (2001 - 2016) | Methods for estimating vaccine coverage rates from [7]. We use the coverage estimates from the untargeted, unbiased vaccination scenario and estimate the proportion of the population susceptible to yellow fever as one minus the vaccine coverage. Assumed 2017 and 2018 identical to 2016 for model estimates of 2017 and 2018. |

Table S2: Data sources for species distribution model, including information on the spatial resolution and range, temporal cadence and range, and use of the data.

| Name | Source | Spatial Resolution (Spatial Range) | Temporal Cadence (Temporal Range) | Use |
|---|---|---|---|---|
| Land surface temperature | MODIS MYD11A1 V006 [8] | 1000 m (global) | 1 day (Mar 2000 - Dec 2018) | Calculated yearly minimum, median, and maximum temperature and for each pixel and averaged over 2001-2017. NOTE: Used MODIS/006/MYD11A1 image collection available on Google Earth Engine. |
| Precipitation | CHIRPS Daily (version 2.0) [9] | 0.05 arc degrees (quasi-global) | 1 day (Jan 1981 - Oct 2018) | Calculated following 3 variables: (1) Yearly total precipitation averaged over 2001-2017. (2) Precipitation of the driest month averaged over 2001-2017. (3) Precipitation of the wettest month averaged over 2001-2017. NOTE: Used UCSB-CHG/CHIRPS/DAILY image collection available on Google Earth Engine. |
| Elevation | NOAA ETOPO1 [10] | 1 arc minute (global) | static (NA) | Used bedrock elevation in meters. NOTE: Used NOAA/NGDC/ETOPO1 image available on Google Earth Engine. |
| Forest Cover | Hansen Global Forest Change v1.5 [11] | 1 arc second (global) | static (NA) | Used percent forest cover from 2000. NOTE: Used UMD/hansen/global_forest_change_2017_v1_5 image available on Google Earth Engine. |
| Land Cover | MODIS MCD12Q1 V006 [12] | 500 meters (global) | yearly (2001 - 2016) | Used FAO-LCCS2 land use layer from 2007. NOTE: Used MODIS/006/MCD12Q1 image collection available on Google Earth Engine. |
| Environmental Vegetation Index | MODIS MOD13A2 V006 [13] | 1000 meters (global) | 16 days (Feb 2000 - Nov 2018) | Calculated median annual EVI averaged over 2001 - 2017. NOTE: Used MODIS/006/MOD13A2 image collection available on Google Earth Engine. |

the capture, resulting in 55 papers (9–63). For the sampling-bias correction, we used Global Biodiversity Information Facility occurrence records from other mosquito species (64), and used locations of other mosquito captures where no occurrence records existed of *Hg. janthinomys*, *Hg. leucocelaunus*, or *Sa. chloropterus* as pseudo-absence points.

#### S2.1.3 Results

The model with the lowest AICc had a regularization parameter of 6.0, and included linear and quadratic features. These were the parameters used to fit the final model. Partial dependence plots showing the marginal response of yellow fever spillover to all covariates are shown in Figure S1. The predicted distribution of yellow fever vectors over all of South America is shown in Figure S2.

### S2.2 Mosquito seasonality

#### S2.2.1 Methods

For each location and vector species, we calculate the maximum monthly mosquito capture, and relative mosquito capture for each month as the percentage of maximum monthly mosquito capture for that location. Similarly, we calculate relative monthly rainfall. We fit a logistic regression of relative mosquito capture on present and lagged relative rainfall using `glm` in R.

#### S2.2.2 Data

For data on mosquito seasonality, we searched the literature and selected papers with field data of adult mosquito captures in consecutive months that also reported rainfall data. We limited to papers that collected at least one of the three sylvatic yellow fever vectors (*Hg. janthinomys*, *Hg. leucocelaenus*, and *Sa. chloropterus*). We identified 6 papers that fit these criteria (52,57,65–68). When data were reported in graphical form, we use WebPlotDigitizer (69) to extract values.

#### S2.2.3 Results

Results from the logistic regression are shown in Table S3. A comparison of model estimates and data are shown in Figure S3.

Table S3: Coefficients from logistic regression of seasonal relative mosquito abundance on current and lagged relative rainfall.

| | Estimate | Std. Error | Z value | p-value |
|---|---|---|---|---|
| Intercept | -2.565475 | 0.4332215 | -5.921855 | 0.0000000 |
| Lagged relative rainfall | 1.996189 | 0.7288357 | 2.738874 | 0.0061650 |
| Current relative rainfall | 1.582762 | 0.7088461 | 2.232871 | 0.0255574 |

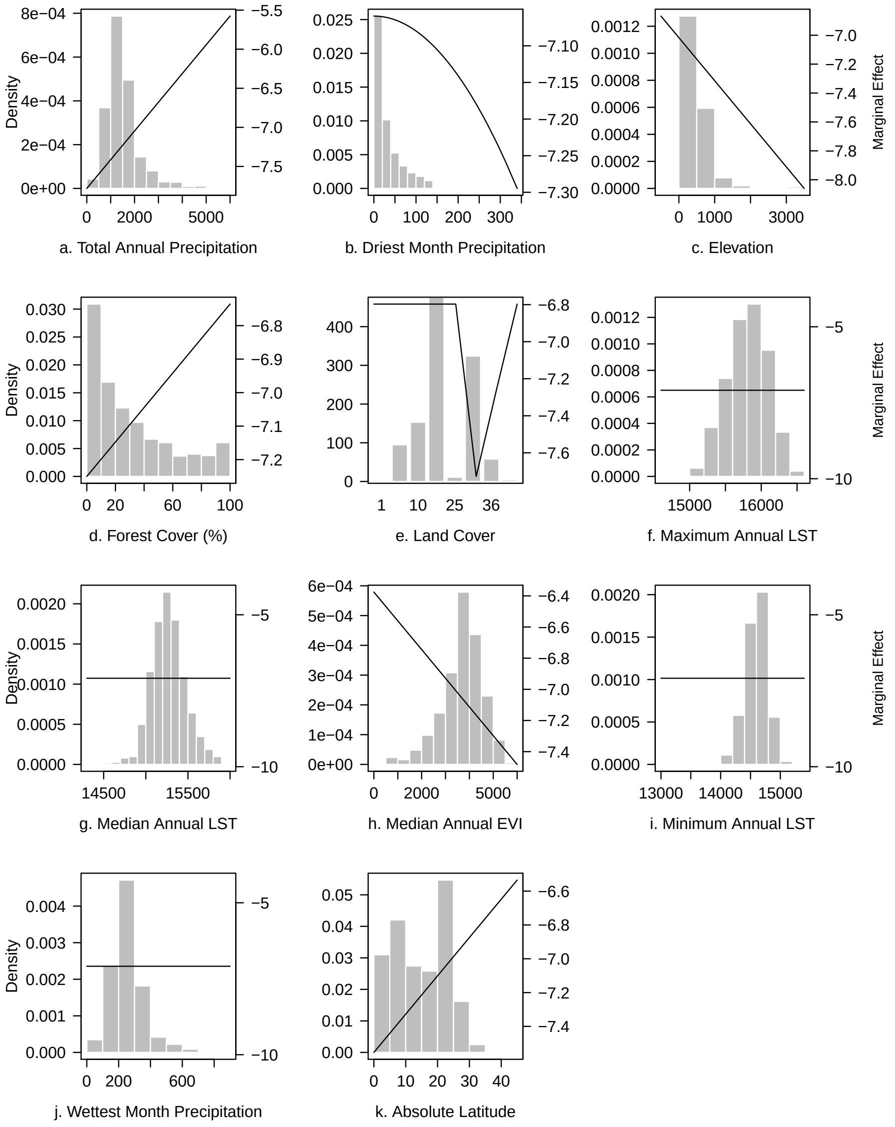


Figure S1: Partial dependence plots of covariates used in species distribution model. Histograms show the distribution of pixels at each covariate value (left axis) and solid lines show the marginal effects of covariate on model prediction (right axis). Covariates with flat marginal effects were identified as unimportant for model prediction. LST Land Surface Temperature, EVI Enhanced Vegetation Index

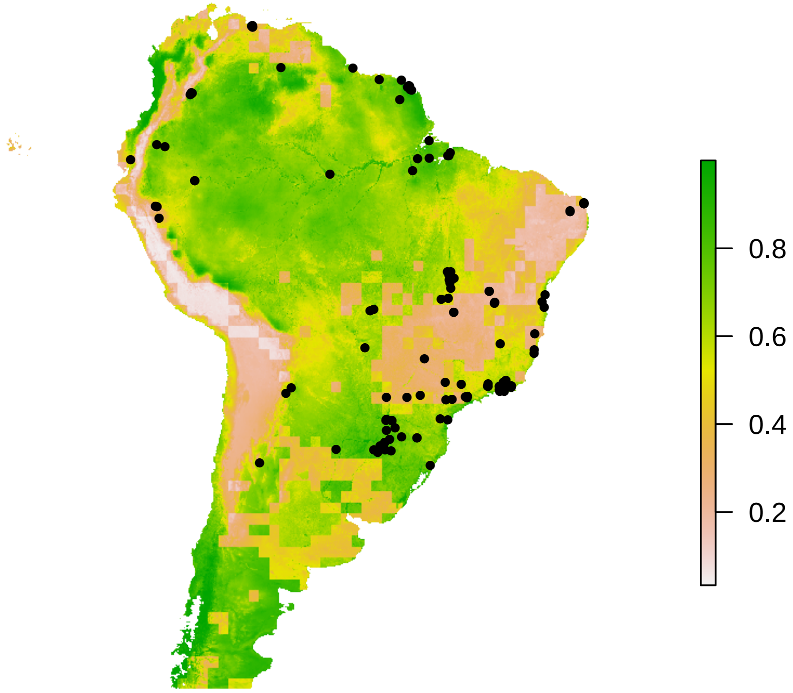


Figure S2: Predicted distribution of vectors from species distribution model, with black points indicating presence locations. Color indicates probability of occurence from highest (green) to lowest (peach).

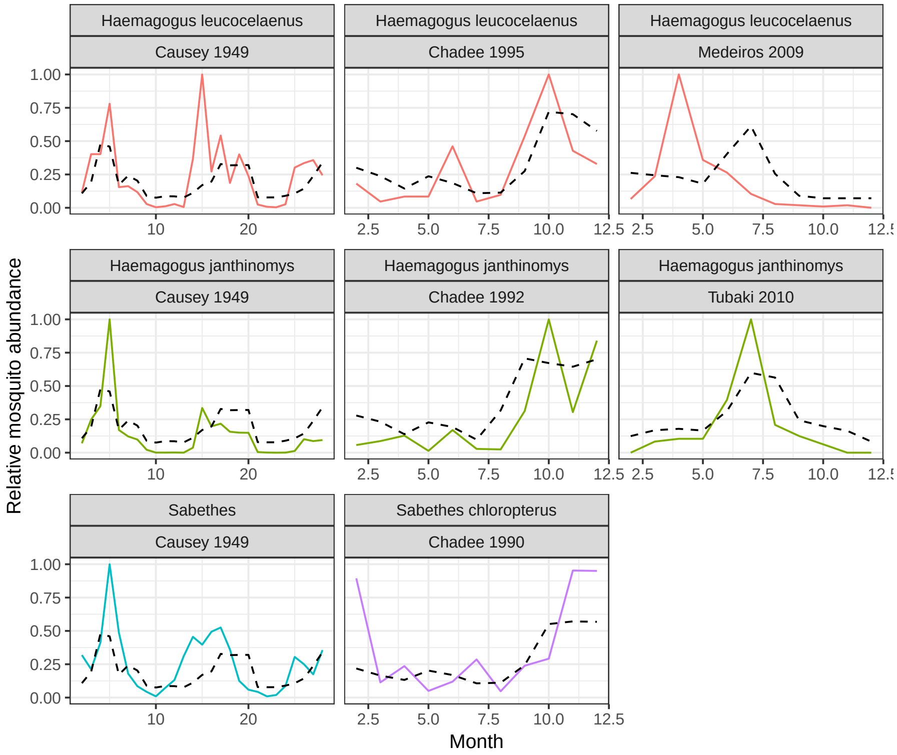


Figure S3: Comparison of mosquito seasonality model to data. Colored lines show data from field studies and dashed black lines show model estimates. Each panel is labeled by the species and study.

## S2.3 Mosquito survival

### S2.3.1 Methods

We fit a quadratic function to the relationship between temperature and lifespan (70,71), with differing coefficients for laboratory and field data given the differing lifespans observed in the two settings:

$$L_i \sim N(c_i(T - T_0)(T - T_m), \sigma^2),$$

where $i$ indicates the setting of lab or field. $L_i$ is the lifespan in each setting, $c_i$ is a scaling coefficient, $T_0$ and $T_m$ are the lower and upper critical thermal limits (respectively), and $T$ is the temperature. This assumes that the critical thermal limits are the same in both laboratory and field settings, but that each setting has different maximum lifespan. We use the coefficient from field data for the mechanistic model in the spillover model. The model is fit using the `rstan` package in R (72), and run with 6 chains with 6000 iterations each. We assume that mosquito mortality is constant at a given temperature due to limited information, and calculate daily survival probability as $p = \exp(-1/L)$.

### S2.3.2 Data

Data are collected from 3 papers (73–75), which were the only sources identified that report *Hg. janthinomys*, *Hg. leucocelaenus*, and *Sa. chloropterus* lifespans and temperatures at which the mosquitoes were reared or caught.

#### S2.3.3 Results

The fitted models for lab and field data are shown in Figure 1e (Main Text).

## S2.4 Mosquito infectiousness

#### S2.4.1 Methods

Given a set of mosquitoes feed upon an infectious blood meal, we assume that the vector competence, or maximum proportion of mosquitoes becoming infectious, is a quadratic function of temperature (71), and that for each mosquito who becomes infectious, the time to infectiousness has a log-normal probability distribution (76):

$$
\begin{aligned}
M &= c(T - T_0)(T - T_m) \\
\mu &= \mu_0 + \mu_T T \\
\sigma &= \exp(\sigma_0 + \sigma_T T) \\
EIP &\sim Log - normal(\mu, \sigma),
\end{aligned}
$$

where $M$ is the maximum proportion infectious, $EIP$ is the time for a mosquito to become infectious, $T$ is the temperature, $T_0$ and $T_m$ are the lower and upper critical thermal limits (respectively), and $c < 0$ is a scaling coefficient. Additionally, $\mu$ is the log of $EIP_{50}$ (time to 50% of max infectious), $\mu_0$ is a scaling factor, and $\mu_T$ is the effect of temperature of $EIP_{50}$. At any point, we model a mosquito's probability of being infectious as $M$ times the cumulative distribution of EIP. The data collected vary in the number of mosquitoes used in each experiment, and mosquitoes were often grouped for biting on primates, so for observations with transmission, we model each censored observation as the probability that at least one mosquito of the group became infectious during the interval and for observations without transmission, we model the censored observation as the probability that none of the mosquitoes became infectious by that time. The model was fit in R using the package `rstan`(72). We run 4 chains with 4000 post-warmup draws per chain and use the median for the parameter estimates.

#### S2.4.2 Data

We collect data (77–83) on yellow fever virus transmission experiments with *Sabethes* and *Haemagogus* species mosquitoes. We use only experiments where mosquito infectivity is tested through bite on a vertebrate. Experimental observations were treated as censored, that is, we either had an interval during which the mosquito or group of mosquitoes became infectious or an interval on which the mosquito or group of mosquitoes did not become infectious during testing (76).

#### S2.4.3 Results

Figure S4 shows transmission experiment data and estimated curves at different temperatures with data. Figure S5 shows vector competence, $EIP_{50}$, and the standard deviation of the log-normal distribution as a function of temperature.

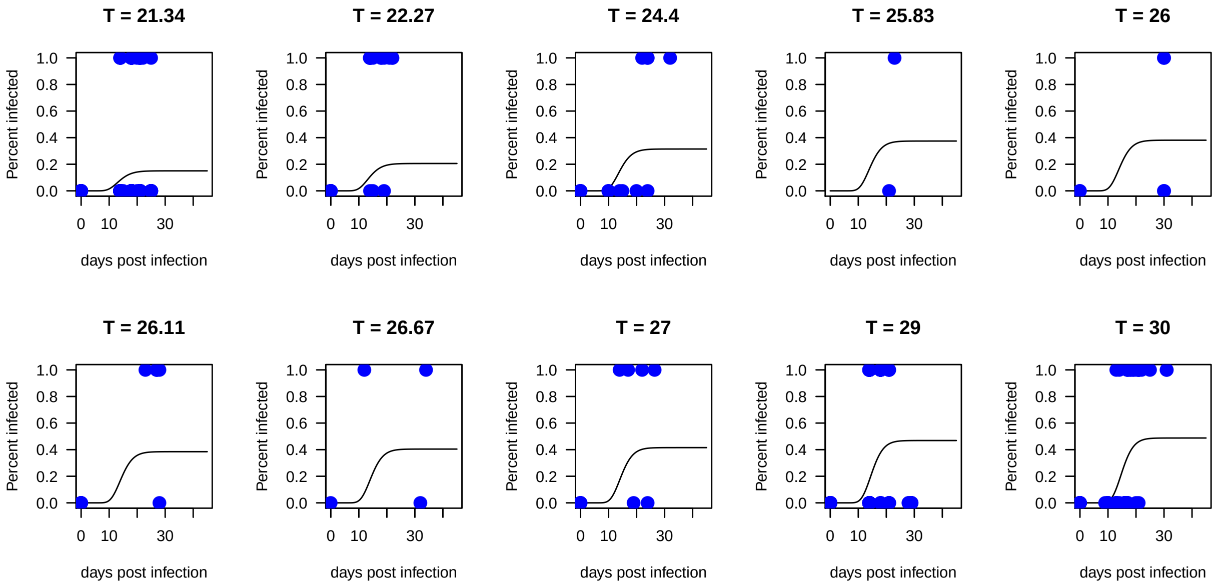


Figure S4: At each temperature where experiments were performed, we plot observations of mosquito biting that resulted in transmission (1) or no transmission (0) on the y-axis and number of days post infectious blood meal on the x-axis in blue points. The black solid line is the modeled probability of mosquito infectiousness, which takes into account both vector competence and a log-normally distributed EIP. Each panel is labeled by the temperature it represents in degrees Celsius.

## S2.5 Mosquito dispersal

### S2.5.1 Methods

We use data on mosquito dispersal from a mark-recapture study, and extract capture station locations using WebPlotDigitizer (69). We then fit a negative exponential dispersal kernel (84), with a negative binomial measurement process:

$$Y_i \sim NB\left(T_i \gamma \frac{1}{2\pi\beta} \exp\left(-\frac{r_i}{\beta}\right), k\right),$$

where $Y_i$ is the number of mosquitoes caught at location $i$, $T_i$ is the amount of time spent capturing at location $i$, $r_i$ is the distance of location $i$ from the release location, $\beta > 0$ is the dispersal scaling parameter, $k > 0$ is the overdispersion parameter, and $\gamma > 0$ is a scaling factor to account for both the number of mosquitoes released and the recapture rate. The model is fit using a Bayesian framework in R using the package `rstan`(72). We run 4 chain with 2000 post-warmup draws per chain and use the median for the parameter estimate.

### S2.5.2 Data

The data used are from a mark-recapture study performed by Causey (85).

### S2.5.3 Results

Figure S6 compares the mark-recapture dispersal data with the model estimates, and incorporates the time spent in collection at each location.

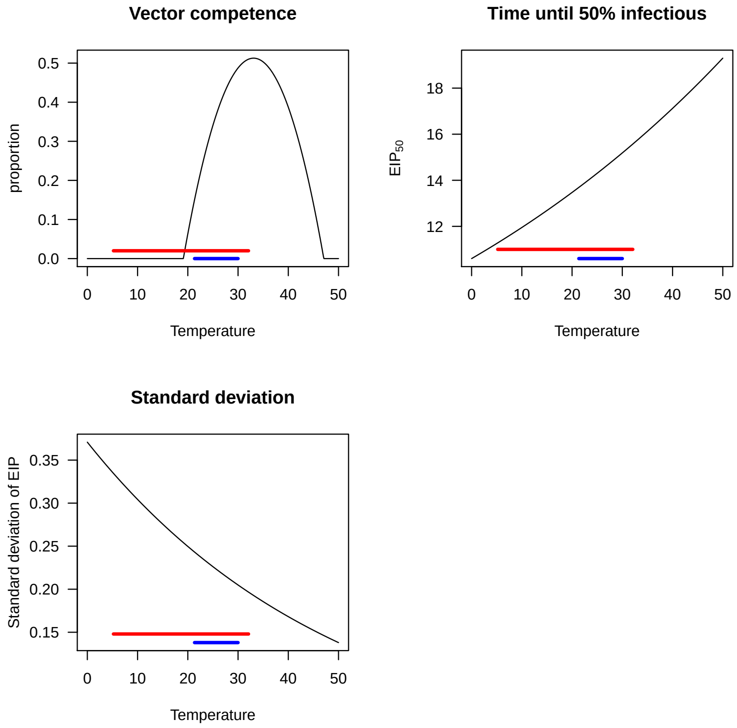


Figure S5: Each of the parameters govering mosquito infectiousness is modeled as temperature dependent. Vector competence determines the horizontal asymptote of mosquito infectiousness, time to 50% infectious determines the point at which mosquito infectiousness is 50% of the way to vector competence, and standard deviation is the the standard deviation of the exponent of the log-normal distrbution. Solid black lines show model estimates. Blue bars show the range of observed temperatures in lab studies and red bars show range of monthly average temperatures observed in Brazil.

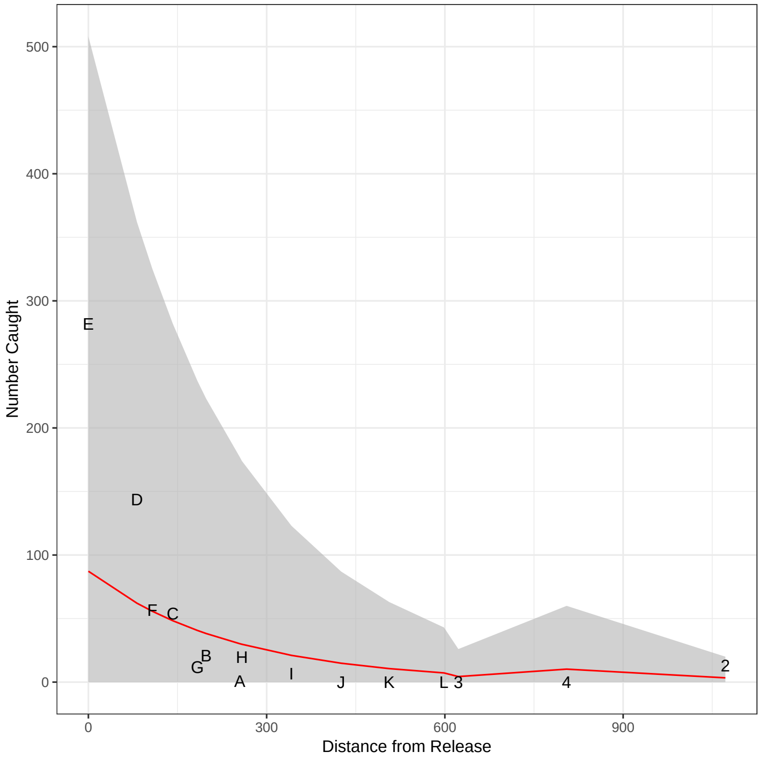


Figure S6: Comparison of dispersal kernel estimate to data. Points are labaled by their trapping location letter from [14]. Red line indicates estimated number of mosquitoes caught at each location from the negative exponential dispersal model when accounting for trapping effort in each sampling location. 95% confidence interval from negative binomial distribution shown in grey shading.

# S3 Phenomenological primate dynamics details

## S3.1 Methods

We fit a phenomenological sine curve with a seven year period (86) to the yearly number of municipality-months with spillover, and then rescale the curve to be between zero and one, as it represents the reservoir infection prevalence. Using the fact that $A\sin(x+B) = a\sin(x) + b\cos(x)$, we assume that human spillover events are a proxy for infection prevalence during reservoir epizootics and fit a linear model to predict number of municipality-months with spillover each year from the sine and cosine of year, transformed to have a 7 year period.

## S3.2 Data

We use monthly human cases of yellow fever from the Brazilian Ministry of Health (87). These cases are reported by municipality of infection and month of first symptoms. We consider spillover to have occurred in a municipality-month if at least one case of yellow fever was reported to have originated from that municipality-month. For the purposes of the phenomenological primate dynamics, we sum the number municipality-month reporting spillover for each year.

## S3.3 Results

The sinusoidal curve explains 52% of the variation in number of municipality-months with spillover (Main text, Figure 2k).

# S4 Model-data comparison details

## S4.1 Methods

Given the multiple hypotheses, we use the Bonferroni procedure (88) to ensure that the family-wise error rate remains 5% over all 17 hypotheses tested (8 for associations between spillover probability and 9 for associations with number of cases given that spillover occurred.) For each hypothesis tested, we report the adjusted p-value $\min(mp_i, 1)$, where $m$ is the total number of hypotheses and $p_i$ is the p-value from the hypothesis.

## S4.2 Data

We used the same data of human cases of yellow fever by municipality and month as described above (Phenomenological primate dynamics: Data). We used Brazilian municipality shapefiles from Instituto Brasileiro de Geografia e Estatística (IBGE) (89) for extracting municipality maximum and mean risk metrics.

## S4.3 Results

See Table S4 for AIC from logistic regression of spillover on risk estimates and AUC from spillover predicted by risk estimates. See Table S5 for results from linear regression of number of reported cases of yellow fever spillover in locations where spillover occurred predicted by model risk estimates and vaccine coverage and Spearman's correlations between risk estimates and number of cases.

Table S4: Model-spillover comparison results. AIC, logistic regression coefficient, logistic regression p-value, and Bonferroni adjusted logistic regression p-value are all reported from a logistic regression of spillover on model estimates and AUC is reported from the receiver operating characteristic curve from predicting spillover with model estimates.

| Risk Metric | Municipality summary | AUC | AIC | Logistic regression coefficient | Logistic regression p-value | Bonferroni adjusted logistic regression p-value |
|---|---|---|---|---|---|---|
| Environmental | mean | 0.705 | 2771.138 | 76.516 | 0.000 | 0.000 |
| Environmental | max | 0.719 | 2735.158 | 18.287 | 0.000 | 0.000 |
| Periodic | mean | 0.776 | 2764.508 | 101.575 | 0.000 | 0.000 |
| Periodic | max | 0.792 | 2731.526 | 22.925 | 0.000 | 0.000 |
| Immunological | mean | 0.597 | 2800.727 | 102.017 | 0.014 | 0.241 |
| Immunological | max | 0.637 | 2786.488 | 27.825 | 0.000 | 0.000 |
| Population-scaled | mean | 0.518 | 2805.175 | -0.049 | 0.704 | 1.000 |
| Population-scaled | max | 0.639 | 2802.825 | 0.003 | 0.019 | 0.322 |

Table S5: Model-case comparison results. Number of reported cases given spillover predicted by risk metrics and vaccine coverage. We also calculate Spearman's rank correlation coefficient for number of cases and risk metrics.

| Risk Metric | Municipality summary | R-squared | Adjusted R-squared | Coefficient | p-value | Bonferroni adjusted p-value | Spearman correlation coefficient |
|---|---|---|---|---|---|---|---|
| Environmental | mean | 0.011 | 0.004 | -43.273 | 0.212 | 1 | 0.008 |
| Environmental | max | 0.020 | 0.012 | -7.408 | 0.098 | 1 | 0.009 |
| Periodic | mean | 0.008 | 0.001 | -47.538 | 0.277 | 1 | 0.011 |
| Periodic | max | 0.015 | 0.008 | -8.076 | 0.145 | 1 | 0.012 |
| Immunological | mean | 0.000 | -0.007 | -23.236 | 0.872 | 1 | 0.004 |
| Immunological | max | 0.004 | -0.003 | -13.085 | 0.465 | 1 | 0.005 |
| Population-scaled | mean | 0.001 | -0.006 | -0.194 | 0.648 | 1 | 0.001 |
| Population-scaled | max | 0.008 | 0.001 | -0.009 | 0.286 | 1 | 0.006 |
| Vaccine Coverage | mean | 0.018 | 0.011 | -2.026 | 0.108 | 1 | 0.010 |

# S5 Boosted regression tree

## S5.1 Methods

We split the data into training (80%) and test (20%) sets using spatially and temporally balanced sampling with the `BalancedSampling` package in R (90). We fit a boosted regression tree to predict spillover for each municipality-month. We consider tree complexities ranging from 1 to 10, and learning rates of 0.005 and 0.001 and for each pair of parameters identified the number of trees (up to 5000) that minimized cross validation predictive deviance (91). We used the `dismo`, `gbm`, and `pdp` packages for the analysis (92–94).

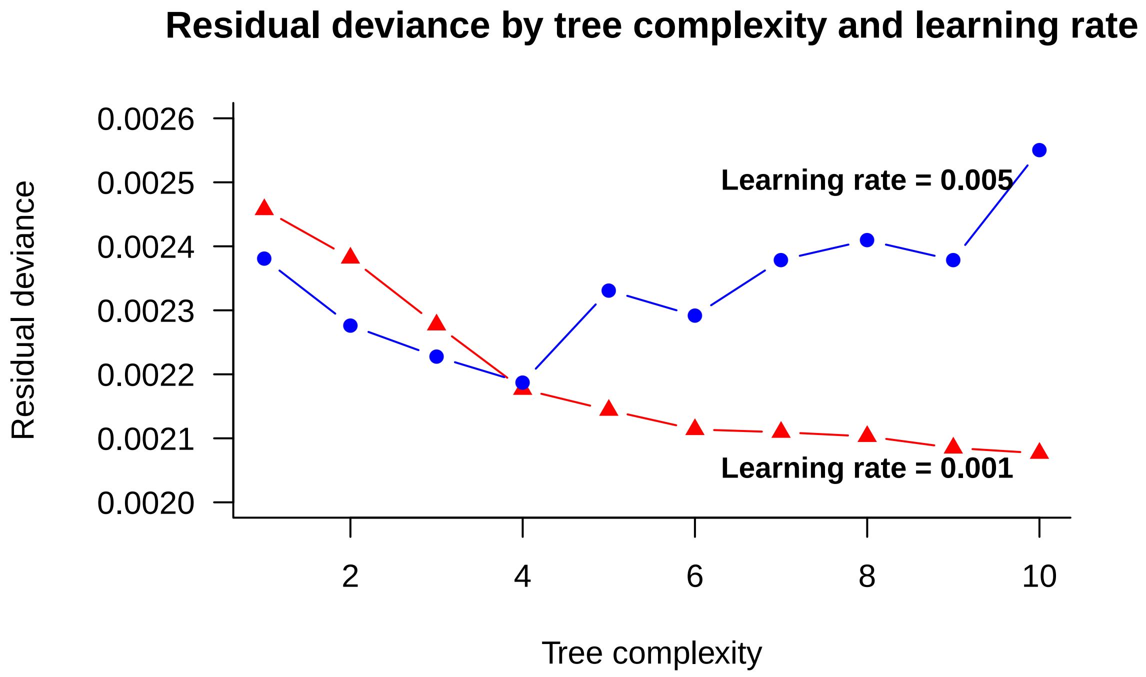


Figure S7: Comparison of predictive deviance across tree complexity and learning rate. Cross validation residual deviance was used to select the optimal number of trees up to 5000 for each set of parameters.

## S5.2 Data

In addition to lagged and current maximum environmental risk, month of the year, region, vaccine coverage and phenomenological primate dynamics (described previously), we use data on current and lagged fire area, maximum and mean primate species richness in the municipality, population density, fire percent, mean air temperature, and monthly precipitation, as described in Table S6. The same data sources are used for primate species distribution, air temperature, precipitation, and vaccine coverage in both the mechanistic and boosted regression tree models (Table S6 and S1), however the data are used differently due to the differing scales of the models (monthly-pixel for mechanistic and monthly-municipality for boosted regression tree) and differing model forms. For the boosted regression tree, we calculate municipality-month averages of the covariates. We use a different data source for human population distribution in the mechanistic model (CEISIN Gridded Population of the World Version 4, UN-Adjusted Population Count) and boosted regression tree analysis (IBGE municipality population estimates), due to the differing scales required for each model. We capitalized on the non-parametric form of the boosted regression tree to include fire area as an additional covariate that could not be included in the mechanistic model due to limited understanding of the mechanism by which land-use influences spillover risk.

## S5.3 Results

For comparison of predictive deviance across different tree complexity and learning rate parameters, see Table S7. This comparison can also be seen visually in Figure S7. The set of parameters that minimized cross validation deviance (tree complexity = 10, learning rate = 0.005, number of trees = 5000) was used as the final model. We also show a partial dependence plot for all variables in Figure S8.

Table S6: Data sources for boosted regression tree analysis, including information on the spatial resolution and range, temporal cadence and range, and use of the data.

| Name | Source | Spatial Resolution (Spatial Range) | Temporal Cadence (Temporal Range) | Use |
|---|---|---|---|---|
| Population Density | IBGE [15] | Municipality (Brazil) | Yearly (2001 - 2016) | We use municipality population estimates and shapefiles of municipalities to determine population density in each municipality. |
| Primate Species Richness | IUCN [2] | NA (global) | static (NA) | Used IUCN species shapefiles to calculate the maximum and spatial average number of primate species in each municipality. Calculations performed in Google Earth Engine [16]. |
| Air temperature | GLDAS-2.1 [6] | 0.25 arc degrees (Global) | 3 hours (Jan 2001 - Oct 2018) | Average temporally then spatially to municipality monthly average air temperature. NOTE: Used NASA/GLDAS/V021/NOAH/ G025/T3H image collection available on Google Earth Engine and performed calculation in Google Earth Engine [16]. |
| Precipitation | TRMM 3B43 [4] | 0.25 arc degrees (Global) | Monthly (Jan 1998 - Sep 2018) | Averaged spatially to get municipality average precipitation. NOTE: Used TRMM/3B43V7 image collection available on Google Earth Engine and performed calculation in Google Earth Engine [16]. |
| Fire Area | MODIS MCD64A1 V006 [17] | 500 meters (Global) | Monthly (Nov 2000 - Oct 2018) | We calculate total area of pixels identified as burned for each month. We also calculate percent fire area by dividing by municipality area. NOTE: Used MODIS/006/MCD64A1 image collection available on Google Earth Engine and performed calculation in Google Earth Engine [16]. |
| Vaccine coverage | Freya Shearer (personal communication) | Municipality (South America and Africa) | yearly (2001 - 2016) | Methods for estimating vaccine coverage rates from [7]. We use the coverage estimates from the untargeted, unbiased vaccination scenario and estimate the proportion of the population susceptible to yellow fever as one minus the vaccine coverage. |

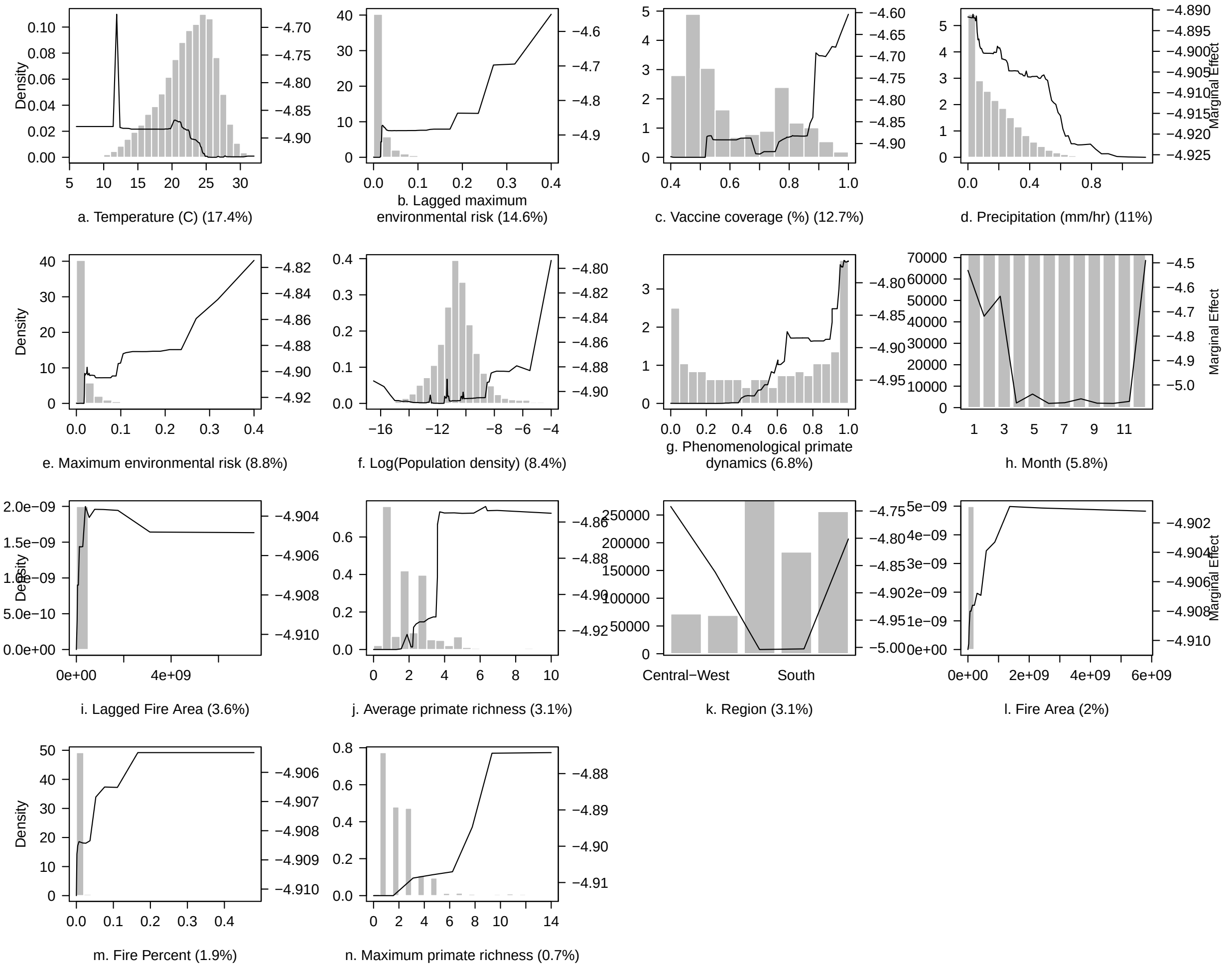


Figure S8: Partial dependence plots of all variables included in the boosted regression tree analysis with histograms showing the distribution of each covariate (left axis) and solid black line showing marginal effect of covariate on model prediction of spillover (right axis).

Table S7: Comparison of predictive deviance across boosted regression tree parameters.

| Tree complexity | Learning rate | Number of trees | Predictive deviance |
|---:|---:|---:|---:|
| 10 | 0.001 | 5000 | 0.0020767 |
| 9 | 0.001 | 5000 | 0.0020850 |
| 8 | 0.001 | 5000 | 0.0021030 |
| 7 | 0.001 | 5000 | 0.0021095 |
| 6 | 0.001 | 5000 | 0.0021138 |
| 5 | 0.001 | 5000 | 0.0021440 |
| 4 | 0.001 | 5000 | 0.0021767 |
| 4 | 0.005 | 4600 | 0.0021869 |
| 3 | 0.005 | 5000 | 0.0022276 |
| 2 | 0.005 | 5000 | 0.0022764 |
| 3 | 0.001 | 5000 | 0.0022773 |
| 6 | 0.005 | 1500 | 0.0022916 |
| 5 | 0.005 | 900 | 0.0023311 |
| 7 | 0.005 | 900 | 0.0023785 |
| 9 | 0.005 | 900 | 0.0023785 |
| 1 | 0.005 | 5000 | 0.0023805 |
| 2 | 0.001 | 5000 | 0.0023818 |
| 8 | 0.005 | 900 | 0.0024094 |
| 1 | 0.001 | 5000 | 0.0024575 |
| 10 | 0.005 | 1050 | 0.0025501 |

# S6 References

## S6.1 Table References


[1] Dimiceli C, Carroll M, Sohlberg R, Kim DH, Kelly M, Townshend JRG. MOD44B MODIS/Terra Vegetation Continuous Fields Yearly L3 Global 250m SIN Grid V006 [Data set]; 2015. https://doi.org/10.5067/MODIS/MOD44B.006.

[2] IUCN. The IUCN Red List of Threatened Species. Version 2018-2.; 2018. http://www.iucnredlist.org.

[3] Hamrick PN, Aldighieri S, Machado G, Leonel DG, Vilca LM, Uriona S, et al. Geographic patterns and environmental factors associated with human yellow fever presence in the Americas. PLoS Neglected Tropical Diseases. 2017;11(9):1–27.

[4] Tropical Rainfall Measuring Mission Project (TRMM). TRMM/TMPA 3B43 TRMM and Other Sources Monthly Rainfall Product V7. Goddard Space Flight Center Distributed Active Archive Center (GSFC DAAC); 2011. http://disc.gsfc.nasa.gov/datacollection/TRMM_3B43_V7.shtml.

[5] Center for International Earth Science Information Network - CIESIN - Columbia University. Gridded Population of the World, Version 4 (GPWv4): Population Count Adjusted to Match 2015 Revision of UN WPP Country Totals.; 2016.

[6] Rodell M, Houser PR, Jambor U, Gottschalck J, Mitchell K, Meng CJ, et al. The Global Land Data Assimilation System. Bull Amer Meteor Soc. 2004;85(3):381–394.

[7] Shearer FM, Moyes CL, Pigott DM, Brady OJ, Marinho F, Deshpande A, et al. Global yellow fever vaccination coverage from 1970 to 2016: An adjusted retrospective analysis. The Lancet Infectious Diseases. 2017;3099(17):1–9.

[8] Wan Z, Hook S, Hulley G. MYD11A1 MODIS/Aqua Land Surface Temperature/Emissivity Daily L3 Global 1km SIN Grid V006 [Data set]. NASA EOSDIS LP DAAC; 2015. https://doi.org/10.5067/MODIS/MYD11A1.006.

[9] Funk C, Peterson P, Landsfeld M, Pedreros D, Verdin J, Shukla S, et al. The climate hazards infrared precipitation with stations—a new environmental record for monitoring extremes. Scientific Data. 2015;.

[10] Amante C, Eakins BW. ETOPO1 1 Arc-Minute Global Relief Model: Procedures, Data Sources and Analysis; 2009.

[11] Hansen MC, Potapov PV, Moore R, Hancher M, Turubanova SA, Tyukavina A, et al. High-Resolution Global Maps of 21st-Century Forest Cover Change. Science. 2013;342:850–53.

[12] Friedl M, Sulla-Menashe D. MCD12Q1 MODIS/Terra+Aqua Land Cover Type Yearly L3 Global 500m SIN Grid V006 [Data set]. NASA EOSDIS Land Processes DAAC; 2015.

[13] Didan K. MOD13A2 MODIS/Terra Vegetation Indices 16-Day L3 Global 1km SIN Grid V006 [Data set]. NASA EOSDIS LP DAAC; 2015.

[14] Causey OR, Kumm HW. Dispersion of forest mosquitoes in Brazil; preliminary studies. The American journal of tropical medicine and hygiene. 1948 may;28(3):469–80. Available from: http://www.ncbi.nlm.nih.gov/pubmed/18859835.

[15] Instituto Brasileiro de Geografia e Estatística. Estatísticas; 2016. https://downloads.ibge.gov.br/downloads_estatisticas.htm.

[16] Gorelick N, Hancher M, Dixon M, Ilyushchenko S, Thau D, Moore R. Google Earth Engine: Planetary-scale geospatial analysis for everyone. Remote Sensing of Environment. 2017;Available from: https://doi.org/10.1016/j.rse.2017.06.031.

[17] Giglio L, Justice C, Boschetti L, Roy D. MCD64A1 MODIS/Terra+Aqua Burned Area Monthly L3 Global 500m SIN Grid V006 [Data set]. NASA EOSDIS Land Processes DAAC; 2015.

## S6.2 Main Text References

1. Gorelick N, Hancher M, Dixon M, Ilyushchenko S, Thau D, Moore R. Google earth engine: Planetary-scale geospatial analysis for everyone. Remote Sensing of Environment [Internet]. 2017; https://doi.org/10.1016/j.rse.2017.06.031

2. Elith J, Phillips SJ, Hastie T, Dudík M, Chee YE, Yates CJ. A statistical explanation of MaxEnt for ecologists. Diversity and Distributions. 2011;17(1):43–57. https://doi.org/10.1111/j.1472-4642.2010.00725.x

3. Phillips S. Maxnet: Fitting 'maxent' species distribution models with 'glmnet' [Internet]. 2017. Retrieved from https://CRAN.R-project.org/package=maxnet

4. Phillips SJ, Anderson RP, Dudík M, Schapire RE, Blair ME. Opening the black box: an open-source release of Maxent. Ecography. 2017;40(7):887–93. https://doi.org/10.1111/ecog.03049

5. Fox SJ, Bellan SE, Perkins TA, Johansson MA, Meyers LA. Downgrading disease transmission risk estimates using terminal importations. bioRxiv [Internet]. 2018; https://doi.org/10.1101/265942

6. GBIF.org. GBIF Occurrence Download [Internet]. 2018. https://doi.org/10.15468/dl.ozsvnj

7. GBIF.org. GBIF Occurrence Download [Internet]. 2018. https://doi.org/10.15468/dl.1uo4ty

8. GBIF.org. GBIF Occurrence Download [Internet]. 2018. https://doi.org/10.15468/dl.gxbxtq

9. Alencar J, Gleiser RM, Morone F, Mello CF de, Silva JS dos, Serra-Freire NM, et al. A comparative study of the effect of multiple immersions on Aedini (Diptera: Culicidae) mosquito eggs with emphasis

on sylvan vectors of yellow fever virus. Memorias do Instituto Oswaldo Cruz. 2014;109(1):114–7. https://doi.org/10.1590/0074-0276130168

10. Aragão NC, Müller GA, Balbino VQ, Costa Junior CRL, Figueirêdo Júnior CS, Alencar J, et al. A list of mosquito species of the Brazilian State of Pernambuco, including the first report of Haemagogus janthinomys (Diptera: Culicidae), yellow fever vector and 14 other species (Diptera: Culicidae). Revista da Sociedade Brasileira de Medicina Tropical. 2010;43(4):458–9. https://doi.org/10.1590/S0037-86822010000400024

11. Pecor JE, Jones J, Turell MJ, Fernandez R, Carbajal F, O'Guinn M, et al. Annotated checklist of the mosquito species encountered during arboviral studies in Iquitos, Peru (Diptera: Culicidae). Journal of the American Mosquito Control Association. 2000;16(3):210–8.

12. Rossi GC. Annotated checklist, distribution, and taxonomic bibliography of the mosquitoes (Insecta: Diptera: Culicidae) of Argentina. Check List. 2015;11(4). https://doi.org/10.15560/11.4.1712

13. Méndez-López MR, Attoui H, Florin D, Calisher CH, Florian-Carrillo JC, Montero S. Association of vectors and environmental conditions during the emergence of Peruvian horse sickness orbivirus and Yunnan orbivirus in northern Peru. Journal of Vector Ecology. 2015;40(2):355–63. https://doi.org/10.1111/jvec.12174

14. Silva JDS, Pacheco JB, Alencar J, Guimarães AÉ. Biodiversity and influence of climatic factors on mosquitoes (Diptera: Culicidae) around the Peixe Angical hydroelectric scheme in the state of Tocantins, Brazil. Memorias do Instituto Oswaldo Cruz. 2010;105(2):155–62. https://doi.org/10.1590/S0074-02762010000200008

15. Marassá AM, Paula MB, Gomes AC, Consales CA. Biotin-avidin sandwich ELISA with specific human isotypes IgG1 and IgG4 for culicidae mosquito blood meal identification from an epizootic yellow fever area in Brazil. Journal of Venomous Animals and Toxins Including Tropical Diseases. 2009;15(4):696–706. https://doi.org/10.1590/S1678-91992009000400008

16. Forattini OP, Gomes AD. Biting Activity of Aedes-Scapularis (Rondani) and Haemagogus Mosquitos in Southern Brazil (Diptera, Culicidae). Revista de saude publica. 1988;22(2):84–93. https://doi.org/10.1590/S0034-89101988000200003

17. Muller GA, Dalavequia MA, Wagner G, Marcondes CB. Blood sucking Diptera (Culicidae, Psychodidae, Simuliidae) in forest fragment under impact of dam in the borderland of Rio Grande do sul and Santa Catarina states, Brazil. Ciencia Rural. 2014;44(7):1194–6. https://doi.org/10.1590/0103-8478cr20131656

18. Inácio CLS, Da Silva JHT, De Melo Freire RC, Gama RA, Marcondes CB, De Melo Ximenes M, et al. Checklist of mosquito species (Diptera: Culicidae) in the Rio Grande do Norte State, Brazil-Contribution of Entomological Surveillance. Journal of Medical Entomology. 2017;54(3):763–73. https://doi.org/10.1093/jme/tjw236

19. Barbosa AA, Navarro-Silva MA, Calado D. Culicidae activity in a restrict forest inside Curitiba urban area (Parana, Brazil). Revista Brasileira de Zoologia. 2003;20(1):59–63. https://doi.org/10.1590/S0101-81752003000100007

20. Montes J. Culicidae fauna of Serra da Cantareira, Sao Paulo, Brazil. Revista de saude publica. 2005;39(4):578–84. https://doi.org/10.1590/S0034-89102005000400010

21. D'Oria JM, Martí DA, Rossi GC. Culicidae, province of Misiones, northeastern Argentina. Check List. 2010;6(1):176–9. https://doi.org/10.15560/6.1.176

22. Aragão ADO, Nunes Neto JP, Cruz ACR, Casseb SMM, Cardoso JF, Silva SP da, et al. Description and phylogeny of the mitochondrial genome of Sabethes chloropterus, Sabethes glaucodaemon and Sabethes belisarioi (Diptera: Culicidae). 2018. https://doi.org/10.1016/j.ygeno.2018.03.016

23. Tatila-Ferreira A, Maia D de A, Alencar J. Development of Preimaginal Stages of Haemagogus Leucocelaenus (Diptera: Culicidae) in Laboratory Conditions. Entomological news. 2017;127(2):142–50. https://doi.org/10.3157/021.127.0209

24. Freitas Silva SO, Mello CF de, Figueiro R, Maia D de A, Alencar J. Distribution of the Mosquito Communities (Diptera: Culicidae) in Oviposition Traps Introduced into the Atlantic Forest in the State of

Rio de Janeiro, Brazil. Vector-Borne and Zoonotic Diseases. 2018;18(4):214–21. https://doi.org/10.1089/vbz.2017.2222

25. Alencar J, Mello CF de, Barbosa LS, Gil-Santana HR, Maia DA, Marcondes CB, et al. Diversity of yellow fever mosquito vectors in the Atlantic forest of Rio de Janeiro, Brazil. Revista da Sociedade Brasileira de Medicina Tropical. 2016;49(3):351–6. https://doi.org/10.1590/0037-8682-0438-2015

26. Cardoso JDC, Paula MB de, Fernandes A, Santos E dos, Almeida MAB de, Fonseca DF da, et al. Ecological aspects of mosquitoes (Diptera: Culicidae) in an Atlantic forest area on the north coast of Rio Grande do Sul State, Brazil. Journal of Vector Ecology. 2011;36(1):175–86. https://doi.org/10.1111/j.1948-7134.2011.00155.x

27. Lira-Vieira AR, Gurgel-Goncalves R, Moreira IM, Cavalcanti Yoshizawa MA, Coutinho ML, Prado PS, et al. Ecological aspects of mosquitoes (Diptera: Culicidae) in the gallery forest of Brasilia National Park, Brazil, with an emphasis on potential vectors of yellow fever. Revista da Sociedade Brasileira de Medicina Tropical. 2013;46(5):566–74. https://doi.org/10.1590/0037-8682-0136-2013

28. Rubio-Palis Y, Moreno JE, Bevilacqua M, Medina D, Martínez Á, Cardenas L, et al. Ecological characterization of anophelines and culicines in the indigenous territory of the Lower Caura River, Bolívar State, Venezuela. Boletin de Malariologia y Salud Ambiental. 2010;50(1):95–107.

29. Pinto CS, Confalonieri UEC, Mascarenhas BM. Ecology of Haemagogus sp. and Sabethes sp. (Diptera: Culicidae) in relation to the microclimates of the Caxiuanã National Forest, Pará, Brazil. Memorias do Instituto Oswaldo Cruz. 2009;104(4):592–8. https://doi.org/10.1590/S0074-02762009000400010

30. Alencar J, De Mello VS, Serra-Freire NM, Silva JDS, Morone F, Guimarães AÉ. Evaluation of mosquito (Diptera: Culicidae) species richness using two sampling methods in the hydroelectric reservoir of Simplício, Minas Gerais, Brazil. Zoological Science. 2012;29(4):218–22. https://doi.org/10.2108/zsj.29.218

31. Virgens TM das, Rezende HR, Pinto IS, Falqueto A. Fauna of mosquitoes (Diptera: Culicidae) in Goytacazes national forest and surrounding area, State of Espírito Santo, southeastern Brazil. Biota Neotropica. 2018;18(1). https://doi.org/10.1590/1676-0611-bn-2016-0250

32. Alencar J, Marcondes CB, Serra-Freire NM, Lorosa ES, Pacheco JB, Guimarães AÉ. Feeding Patterns of Haemagogus capricornii and Haemagogus leucocelaenus (Diptera: Culicidae) in Two Brazilian States (Rio de Janeiro and Goiás). Journal of Medical Entomology [Internet]. 2008 Sep;45(5):873–6. https://doi.org/10.1093/jmedent/45.5.873

33. Alencar J, Morone F, Mello CFD, Dégallier N, Lucio PS, Serra-Freire NMD, et al. Flight height preference for oviposition of mosquito (diptera: Culicidae) vectors of sylvatic yellow fever virus near the hydroelectric reservoir of simplicío, minas Gerais, Brazil. Journal of Medical Entomology. 2013;50(4):791–5. https://doi.org/10.1603/ME12120

34. Mucci LF, Medeiros-Sousa AR, Ceretti-Júnior W, Fernandes A, Camargo AA, Evangelista E, et al. Haemagogus leucocelaenus and Other Mosquitoes Potentially Associated with Sylvatic Yellow Fever in Cantareira State Park in the São Paulo Metropolitan Area, Brazil. Journal of the American Mosquito Control Association. 2016;32(4):329–32. https://doi.org/10.2987/16-6587.1

35. Fernandez Z, Richartz R, Da Rosa AT, Soccol VT. Identification of the encephalitis equine virus, Brazil. Revista de saude publica. 2000;34(3):232–5. https://doi.org/10.1590/S0034-89102000000300004

36. Zequi JAC, Lopes J, Medri IM. Immature specimens of Culicidae (Diptera) found in installed recipients in forest fragments in the Londrina, Parana, Brazil. Revista Brasileira de Zoologia. 2005;22(3):656–61. https://doi.org/10.1590/S0101-81752005000300021

37. Santos CF, Borges M. Impact of livestock on a mosquito community (Diptera: Culicidae) in a Brazilian tropical dry forest. Revista da Sociedade Brasileira de Medicina Tropical. 2015;48(4):474–8. https://doi.org/10.1590/0037-8682-0022-2015

38. Alencar J, Serra-Friere NM, Marcondes CB, Silva JS, Correa FF, Guimarães AÉ. Influence of Climatic Factors on the Population Dynamics of Haemagogus Janthinomys (Diptera: Culicidae), a Vector of Sylvatic

Yellow Fever. Entomologival news. 2010;121(1):45–52. https://doi.org/10.3157/021.121.0109

39. Santos CF, Silva AC, Rodrigues RA, Jesus JSR, Borges MAZ. Inventory of mosquitoes (Diptera: Culicidae) in conservation units in Brazilian tropical dry forests. Revista do Instituto de Medicina Tropical de Sao Paulo. 2015;57(3):227–32. https://doi.org/10.1590/S0036-46652015000300008

40. Souza RP de, Petrella S, Coimbra TLM, Maeda AY, Rocco IM, Bisordi I, et al. Isolation of yellow fever virus (YFV) from naturally infected haemagogus (conopostegus) leucocelaenus (diptera, culicidae) in São Paulo state, Brazil, 2009. Revista do Instituto de Medicina Tropical de Sao Paulo. 2011;53(3):133–9. https://doi.org/10.1590/S0036-46652011000300004

41. Vasconcelos PFC, Sperb AF, Monteiro HAO, Torres MAN, Sousa MRS, Vasconcelos HB, et al. Isolations of yellow fever virus from Haemagogus leucocelaenus in Rio Grande do Sul State, Brazil. Transactions of the Royal Society of Tropical Medicine and Hygiene. 2003;97(1):60–2. https://doi.org/10.1016/S0035-9203(03)90023-X

42. Yanoviak SP, Lounibos LP, Weaver SC. Land use affects macroinvertebrate community composition in phytotelmata in the Peruvian Amazon. Annals of the Entomological Society of America. 2006;99(6):1172–81. https://doi.org/10.1603/0013-8746(2006)99[1172:LUAMCC]2.0.CO;2

43. Serra OP, Cardoso BF, Maria Ribeiro AL, Leal dos Santos FA, Slhessarenko RD. Mayaro virus and dengue virus 1 and 4 natural infection in culicids from Cuiaba, state of Mato Grosso, Brazil. Memorias do Instituto Oswaldo Cruz. 2016;111(1):20–9. https://doi.org/10.1590/0074-02760150270

44. Correa FF, Gleiser RM, Leite PJ, Fagundes E, Gil-Santana HR, Mello CF, et al. Mosquito communities in Nova Iguaçu Natural Park, Rio de Janeiro, Brazil. Journal of the American Mosquito Control Association. 2014;30(2):83–90. https://doi.org/10.2987/13-6372.1

45. Orlandin E, Santos EB, Piovesan M, Favretto MA, Schneeberger AH, Souza VO, et al. Mosquitoes (Diptera: Culicidae) from crepuscular period in an Atlantic Forest area in Southern Brazil. Brazilian Journal of Biology. 2017;77(1):60–7. https://doi.org/10.1590/1519-6984.09815

46. Medeiros-Sousa AR, Fernandes A, Ceretti-Junior W, Wilke ABB, Marrelli MT. Mosquitoes in urban green spaces: Using an island biogeographic approach to identify drivers of species richness and composition. Scientific Reports. 2017;7(1). https://doi.org/10.1038/s41598-017-18208-x

47. De Figueiredo ML, De C Gomes A, Amarilla AA, De S Leandro A, De S Orrico A, De Araujo RF, et al. Mosquitoes infected with dengue viruses in Brazil. Virology Journal. 2010;7. https://doi.org/10.1186/1743-422X-7-152

48. Alencar J, Gil-Santana HR, Oliveira R de FN de, Dégallier N, Guimarães AÉ. Natural Breeding Sites for Haemagogus Mosquitoes (Diptera, Culicidae) in Brazil. Entomological News [Internet]. 2010 Sep;121(4):393–6. https://doi.org/10.3157/021.121.0414

49. Chadee DD, Beier JC. Natural variation in blood-feeding kinetics of four mosquito vectors. Journal of Vector Ecology. 1996;21(2):150–5.

50. Linares MA, Laurito M, Visintin AM, Rossi GC, Stein M, Almirón WR. New mosquito records (Diptera: Culicidae) from northwestern Argentina. Check List. 2016;12(4). https://doi.org/10.15560/12.4.1944

51. Müller GA, Kuwabara EF, Duque JE, Navarro-Silva MA, Marcondes CB. New records of mosquito species (Diptera: Culicidae) for Santa Catarina and Paraná (Brazil). Biota Neotropica. 2008;8(4):211–8. https://doi.org/10.1590/S1676-06032008000400021

52. Tubaki RM, Menezes RMTD, Vesgueiro FT, Cardoso RP. Observations on Haemagogus janthinomys Dyar (Diptera: Culicidae) and other mosquito populations within tree holes in a gallery forest in the northwestern region of Sao Paulo state, Brazil. Neotropical entomology. 2010;39(4):664–70. https://doi.org/Doi 10.1590/S1519-566x2010000400030

53. Talaga S, Murienne J, Dejean A, Leroy C. Online database for mosquito (Diptera, Culicidae) occurrence records in French guiana. ZooKeys. 2015;2015(532):107–15. https://doi.org/10.3897/zookeys.532.6176

54. Tatila-Ferreira A, Maia D de A, Santos de Abreu FV, Rodrigues WC, Alencar J. Oviposition behavior

of Haemagogus leucocelaenus (Diptera: culicidae), a vector of wild yellow fever in Brazil. Revista do Instituto de Medicina Tropical de Sao Paulo. 2017;59:UNSP e60–UNSP e60. https://doi.org/10.1590/S1678-9946201759060

55. Pauvolid-Corrêa A, Tavares FN, Alencar J, Silva JDS, Murta M, Serra-Freire NM, et al. Preliminary investigation of culicidae species in South Pantanal, Brazil and their potential importance in arbovirus transmission. Revista do Instituto de Medicina Tropical de Sao Paulo. 2010;52(1):17–23. https://doi.org/10.1590/S0036-46652010000100004

56. Vale Barbosa M das G, Fe NF, Ribera Marciao AH, Thome da Silva AP, Monteiro WM, de Farias Guerra MV, et al. Record of epidemiologically important Culicidae in the rural area of Manaus, Amazonas. Revista da Sociedade Brasileira de Medicina Tropical. 2008;41(6):658–63.

57. Medeiros AS, Marcondes CB, De Azevedo PRM, Jerônimo SMR, Silva V, De De Ximenes M. Seasonal variation of potential flavivirus vectors in an urban biological reserve in Northeastern Brazil. Journal of Medical Entomology. 2009;46(6):1450–7. https://doi.org/10.1603/033.046.0630

58. Dos Santos EB, Orlandin E, Piovesan M, Favretto MA. Short communication on the mosquitoes of a forested urban area at the municipality of Joaçaba, Santa Catarina, Brazil. Entomotropica. 2016;31(2016):91–4.

59. Mangudo C, Aparicio JP, Rossi GC, Gleiser RM. Tree hole mosquito species composition and relative abundances differ between urban and adjacent forest habitats in northwestern Argentina. Bulletin of Entomological Research. 2018;108(2):203–12. https://doi.org/10.1017/S0007485317000700

60. Talaga S, Dejean A, Carinci R, Gaborit P, Dusfour I, Girod R. Updated Checklist of the Mosquitoes (Diptera: Culicidae) of French Guiana. Journal of Medical Entomology. 2015;52(5):770–82. https://doi.org/10.1093/jme/tjv109

61. Alencar J, Mello CF de, Gil-Santana HR, Guimarães AE, Almeida SAS de, Gleiser RM. Vertical oviposition activity of mosquitoes in the Atlantic Forest of Brazil with emphasis on the sylvan vector, Haemagogus leucocelaenus (Diptera: Culicidae). Journal of Vector Ecology. 2016;41(1):18–26. https://doi.org/10.1111/jvec.12189

62. Vasconcelos PFC, Rosa APAT, Rodrigues SG, Rosa EST, Monteiro HAO, Cruz ACR, et al. Yellow fever in Para State, Amazon region of Brazil, 1998-1999: Entomologic and epidemiologic findings. Emerging Infectious Diseases. 2001;7(3):565–9. https://doi.org/10.3201/eid0703.010338

63. Cardoso JC, Almeida MAB de, Santos E dos, Fonseca DF da, Sallum MAM, Noll CA, et al. Yellow fever virus in Haemagogus leucocelaenus and Aedes serratus mosquitoes, Southern Brazil, 2008. Emerging Infectious Diseases. 2010;16(12):1918–24. https://doi.org/10.3201/eid1612.100608

64. GBIF.org. GBIF Occurrence Download [Internet]. 2018. https://doi.org/10.15468/dl.wvs9g2

65. Causey OR, Dos Santos GV. Diurnal Mosquitoes in an Area of Small Residual Forests in Brazil1. Annals of the Entomological Society of America [Internet]. 1949 Dec;42(4):471–82. https://doi.org/10.1093/aesa/42.4.471

66. Chadee DD. Seasonal abundance and diel landing periodicity of Sabethes chloropterus (Diptera: Culicidae) in Trinidad, West Indies. Journal of medical entomology. 1990;27(6):1041–4. https://doi.org/10.1093/jmedent/27.6.1041

67. Chadee DD, Tikasingh ES, Ganesh R. Seasonality, biting cycle and parity of the yellow fever vector mosquito Haemagogus janthinomys in Trinidad. Medical and Veterinary Entomology. 1992;6(2):143–8. https://doi.org/10.1111/j.1365-2915.1992.tb00592.x

68. Chadee DD, Ganesh R, Hingwan JO, Tikasingh ES. Seasonal abundance, biting cycle and parity of the mosquito Haemagogus leucocelaenus in Trinidad, West Indies. Medical and Veterinary Entomology [Internet].

1995 Oct;9(4):372–6. https://doi.org/10.1111/j.1365-2915.1995.tb00006.x

69. Rohatgi A. WebPlotDigitizer [Internet]. 2018. Retrieved from https://automeris.io/WebPlotDigitizer

70. Mordecai EA, Paaijmans KP, Johnson LR, Balzer C, Ben-Horin T, Moor E de, et al. Optimal temperature for malaria transmission is dramatically lower than previously predicted. Ecology Letters. 2013;16(1):22–30. https://doi.org/10.1111/ele.12015

71. Mordecai EA, Cohen JM, Evans MV, Gudapati P, Johnson LR, Lippi CA, et al. Detecting the impact of temperature on transmission of Zika, dengue, and chikungunya using mechanistic models. Althouse B, editor. PLOS Neglected Tropical Diseases [Internet]. 2017 Apr;11(4):e0005568. https://doi.org/10.1371/journal.pntd.0005568

72. Team SD. RStan: The r interface to stan [Internet]. 2018. Retrieved from http://mc-stan.org/

73. Bates M. The Development and Longevity of Haemagogus Mosquitoes under Laboratory Conditions1. Annals of the Entomological Society of America [Internet]. 1947 Mar;40(1):1–12. https://doi.org/10.1093/aesa/40.1.1

74. Galindo P. Bionomics of Sabethes Chloropterus Humboldt, a Vector of Sylvan Yellow Fever in Middle America 1. The American Journal of Tropical Medicine and Hygiene [Internet]. 1958 Jul;7(4):429–40. https://doi.org/10.4269/ajtmh.1958.7.429

75. Dégallier N, SÁ Filho GC, Monteiro HAO, Castro FC, Vaz Da Silva O, Brandão RCF, et al. Release-Recapture Experiments with Canopy Mosquitoes in the Genera Haemagogus and Sabeihes (Diptera: Culicidae) in Brazilian Amazonia. Journal of Medical Entomology [Internet]. 1998 Nov;35(6):931–6. https://doi.org/10.1093/jmedent/35.6.931

76. Johansson MA, Arana-Vizcarrondo N, Biggerstaff BJ, Staples JE. Incubation periods of yellow fever virus. American Journal of Tropical Medicine and Hygiene. 2010;83(1):183–8. https://doi.org/10.4269/ajtmh.2010.09-0782

77. Waddell MB, Taylor RM. Studies on Cyclic Passage of Yellow Fever Virus in South American Mammals and Mosquitoes: Marmosets (Callithrix aurita) and Cebus Monkeys (Cebus versutus) in Combination with Aedes aegypti and Haemagogus equinus. The American Journal of Tropical Medicine and Hygiene [Internet]. 1945 May;s1-25(3):225–30. https://doi.org/10.4269/ajtmh.1945.s1-25.225

78. Roca-Garcia M, Bates M. Laboratory Studies of the Saimiri-Haemagogus Cycle of Jungle Yellow Fever. The American Journal of Tropical Medicine and Hygiene [Internet]. 1945 May;s1-25(3):203–16. https://doi.org/10.4269/ajtmh.1945.s1-25.203

79. Anderson CR, Osorno-Mesa E. The laboratory transmission of yellow fever virus by Haemagogus splendens. The American journal of tropical medicine and hygiene [Internet]. 1946 Sep;26(5):613–8. Retrieved from http://www.ncbi.nlm.nih.gov/pubmed/21003269

80. Waddell MB, Taylor RM. Studies on the Cyclic Passage of Yellow Fever Virus in South American Mammals and MosquitoesI III. Further Observations on Haemagogus equinus as a vector of the virus. The American Journal of Tropical Medicine and Hygiene [Internet]. 1947 Jul;s1-27(4):471–6. https://doi.org/10.4269/ajtmh.1947.s1-27.471

81. Kumm HW, Waddell MB. Haemagogus Capricornii Lutz as a Laboratory Vector of Yellow Fever 1. The American Journal of Tropical Medicine and Hygiene [Internet]. 1948 Mar;s1-28(2):247–52. https://doi.org/10.4269/ajtmh.1948.s1-28.247

82. Waddell MB. Comparative efficacy of certain South American Aëdes and Haemagogus mosquitoes as laboratory vectors of yellow fever. The American journal of tropical medicine and hygiene [Internet]. 1949 Jul;29(4):567–75. Retrieved from http://www.ncbi.nlm.nih.gov/pubmed/18153054

83. Galindo P, Trapido H, Rodaniche E de. Experimental Transmission of Yellow Fever by Central American Species of Haemagogus and Sabethes Chloropterus. The American Journal of Tropical Medicine and Hygiene

[Internet]. 1956 Nov;5(6):1022–31. https://doi.org/10.4269/ajtmh.1956.5.1022

84. Estep LK, Burkett-Cadena ND, Hill GE, Unnasch RS, Unnasch TR. Estimation of Dispersal Distances of Culex erraticus in a Focus of Eastern Equine Encephalitis Virus in the Southeastern United States. Journal of Medical Entomology [Internet]. 2010;47(6):977–86. https://doi.org/10.1603/ME10056

85. Causey OR, Kumm HW. Dispersion of forest mosquitoes in Brazil; preliminary studies. The American journal of tropical medicine and hygiene [Internet]. 1948 May;28(3):469–80. Retrieved from http://www.ncbi.nlm.nih.gov/pubmed/18859835

86. Camara FP, Gomes ALBB, Carvalho LMF de, Castello LGV. Dynamic behavior of sylvatic yellow fever in Brazil (1954-2008). Revista da Sociedade Brasileira de Medicina Tropical [Internet]. 2011 Jun;44(3):297–9. https://doi.org/10.1590/S0037-86822011005000024

87. Ministério da Saúde do Brasil. Epidemiológicas e morbidade. http://www2.datasus.gov.br/DATASUS/index.php?area=0203; 2017.

88. Goeman JJ, Solari A. Multiple hypothesis testing in genomics. Statistics in Medicine. 2014;33(11):1946–78. https://doi.org/10.1002/sim.6082

89. Instituto Brasileiro de Geografia e Estatística. Geociências. https://downloads.ibge.gov.br/downloads_geociencias.htm; 2016.

90. Grafström A, Lisic J. BalancedSampling: Balanced and spatially balanced sampling [Internet]. 2018. Retrieved from https://CRAN.R-project.org/package=BalancedSampling

91. Elith J, Leathwick JR, Hastie T. A working guide to boosted regression trees. Journal of Animal Ecology. 2008;77(4):802–13. https://doi.org/10.1111/j.1365-2656.2008.01390.x

92. Hijmans RJ, Phillips S, Leathwick J, Elith J. Dismo: Species distribution modeling [Internet]. 2017. Retrieved from https://CRAN.R-project.org/package=dismo

93. Greenwell B, Boehmke B, Cunningham J, Developers G. Gbm: Generalized boosted regression models [Internet]. 2018. Retrieved from https://CRAN.R-project.org/package=gbm

94. Greenwell BM. Pdp: An r package for constructing partial dependence plots. The R Journal [Internet]. 2017;9(1):421–36. Retrieved from https://journal.r-project.org/archive/2017/RJ-2017-016/index.html
